# Supplementary material for: Design of Geraniol-Loaded Nanocapsules for Use Against Salmonella Infantis: Evaluation in an In Vitro Poultry Model
Source: Pharmaceutics. 2025 Jun 27;17(7):840. doi: 10.3390/pharmaceutics17070840 (PMC12300759; doi:10.3390/pharmaceutics17070840)
Supplement: Supplementary file 1 [file pharmaceutics-17-00840-s001.zip › pharmaceutics-3681568-supplementary.pdf]

Supplementary Table S1. Experimental design matrix showing the factors, levels and response variables considered in the development of geraniol nanocapsules formulations.

Formulation	Geraniol mcL	Miglyol® 810N mcL	Tween 80:Span80 mcL	Drugcoat E PO mg	Size nm	PDI	Zeta potential mV	EE %
**1**	50	75	75	50	86.1	0.1070	11.2	31.5
**2**	50	50	125	75	149	0.1030	13.5	42.9
**3**	50	50	100	50	122.0	0.0639	12.4	40.6
**4**	50	25	125	50	211.0	0.2220	12.1	34.9
**5**	50	50	75	25	93.5	0.0923	11.7	32.8
**6**	50	25	75	50	195.0	0.0929	14.4	31.0
**7**	50	50	75	75	122.0	0.0873	15.8	16.0
**8**	50	25	100	75	273.0	0.2660	15.2	52.1
**9**	50	75	125	50	89.6	0.1180	11.0	49.2
**10**	50	50	100	50	122.0	0.0791	12.4	38.0
**11**	50	50	125	25	133.0	0.1370	10.3	36.0
**12**	50	75	100	25	73.6	0.1370	10.0	29.0
**13**	50	50	100	50	120.0	0.0609	11.9	41.2
**14**	50	25	100	25	183.0	0.1460	12.0	28.7
**15**	50	75	100	75	85.6	0.0309	13.7	38.4
